# Antecedents of continuance intention in online learning systems among vocational college students: The moderating effect of gender

**DOI:** 10.3389/fpsyg.2022.1088270

**Published:** 2022-12-22

**Authors:** Xia Li, Xiuling Wang, Chenming Wei

**Affiliations:** ^1^School of Teacher Education, Weifang University, Weifang, China; ^2^School of General Education, Weifang University of Science and Technology, Shouguang, China

**Keywords:** intention to continue using online learning, anxiety, computer self-efficacy, social interaction, expectation-confirmation model

## Abstract

**Background:**

The primary objective of this study is to ascertain whether the Expectation Confirmation Model can be expanded by external variables including computer anxiety, social interaction, and self-efficacy to better understand the intention to continue using online learning systems in the post-pandemic era among vocational college students. Moreover, this research argues that the intention to continue using online learning systems among students may be gender-sensitive.

**Methods:**

The researchers surveyed 482 students from eight vocational colleges in Jiangxi Province using a structured questionnaire. Partial Least Squares Structural equation modeling is used to verify the research model.

**Results:**

The outcomes demonstrate that the proposed model adequately explains the continuous use intention for online learning systems at a 76.6% confidence level. All of the newly introduced variables in the ECM are shown to be significant and relevant to explicate continuous use intention. Our survey results show that gender differences in intention to continue using online learning systems exist objectively, but this difference is not a natural difference.

**Conclusion:**

This research fills a void in the current literature on online learning and probes into how learning may be made more long-lasting in intricate environments.

## 1. Introduction

The devastation caused by the COVID-19 pandemic has reached approximately every sector of society around the globe, and vocational colleges are no exception (Xu et al., 2017; Zapata-Cuervo et al., 2022). It has led to the interruption of most traditional teaching methods and compelled both the teaching and learning processes to undergo unpredictable and rapid shifts, such as the traditional teaching and learning activities of more than 1,300 vocational colleges and nearly 11,000 vocational high schools in China have had to be discontinued and transferred to an online model (Han et al., 2021). Accordingly, educational institutions have re-established plans to begin offering online courses in a relatively short amount of time (Persada et al., 2021; Soria-Barreto et al., 2021; Wut et al., 2022). Online learning is a form of synchronous learning in different places that is based on networking technology and realizes cross-border human-computer, interpersonal communication, and long-distance interaction through information technology (Ferreira et al., 2018). Compared to conventional learning, online learning has numerous benefits. For example, firstly, the cost of education is lowered for students because of the widespread availability of high-quality educational resources that may be shared among them through online learning Mushtaque et al. (2022). Secondly, online learning is conducive to communication and cooperation between students and allows them to share their ideas with others in a timely manner (Wut et al., 2022). Thirdly, online learning allows for personalized learning and flexible scheduling of learning progress (Besser et al., 2022). In general, the advantages of online learning are more prominent during the epidemic.

Numerous academics have studied online learning in recent decades from a variety of angles based on Technology Acceptance Model (TAM), Information System Continuance (ISC), and other theories (Karaoglan Yilmaz and Yilmaz, 2020; Karaoğlan Yılmaz, 2022). The majority of these studies concentrate on the adoption and satisfaction of online learning, but García-Morales et al. (2021) argue that the sustainable development and service of online learning in the future will become the focus of post-epidemic education. As a result, the model of expectation confirmation will serve as the foundation for this research, which will investigate the factors that influence vocational college students’ decisions on whether or not they will continue using online learning in the post-epidemic age.

Scholars conducted research on the factors that affect students’ continuous use of online learning in various scenarios (Li and Zhao, 2021; Persada et al., 2021; Soria-Barreto et al., 2021). It is concluded that the interactivity of online learning systems is the key to ensure the quality of online learning and improve the intention to use online learning systems continuously. For instance, Wut et al. (2022) considered that the focus of the future online and offline learning debate is student experience. And interactivity is the most essential element affecting the online learning experience. A learning system with good experience can reduce the cognitive load of students when using the system and improve their intention to use it continuously. Li and Zhao (2021) extended the expectation confirmation model by including social interaction and quality to investigate the factors that influence the intention to continuously use online learning. In addition to studying the external factors of learners, researchers are also increasingly interested in their psychological traits (Karaoglan Yilmaz and Yilmaz, 2020; Karaoglan Yilmaz, 2022; Karaoğlan Yılmaz, 2022; Yilmaz et al., 2022). For example, Self-determination theory (SDT) was used by Luo et al. (2021) to build a model that elaborate the links between students’ basic psychological needs, intrinsic and extrinsic motivation, and continued intention to use online self-regulated learning. Sharma et al. (2022) explored the relationship between self-congruity, perceived enjoyment, and the intention to continue using e-learning. The above literature review demonstrates that there is a close relationship between online learners’ psychological factors and their intention to continue using online learning.

However, few studies have been conducted on the effects of emotional elements like computer self-efficacy and anxiety on online learners’ continued intentions. This could be interpreted as a gap in the existing literature that requires more investigation. Bao (2020) points out that this transition to online learning occurs suddenly, so the degrees of anxiety that learners experience need to be alleviated in order to verify the efficiency of online learning. Meanwhile, the survey reveals that students at vocational colleges have a hard time maintaining focus while studying online for extended periods of time (Panigrahi et al., 2018; Baber, 2021; Soria-Barreto et al., 2021). Consequently, one of the methods to boost the vocational college students’ intention to continue using online learning systems is to enhance learners’ self-efficacy and decrease students’ anxiety level regarding the usage of online learning systems (Niu et al., 2022).

Compared with previous studies, the innovations of this study mainly include the following three points: Firstly, it adapts the expectation confirmation model by introducing three new context-specific elements, namely, computer anxiety, computer self-efficacy, and social interaction. Secondly, most of the prior studies treated samples as a homogenous group (Panigrahi et al., 2018; Chung et al., 2020; Wang S. et al., 2021). However, as the audience of online learning systems is becoming an increasingly diverse group, a differentiated strategy is required to encourage continuous user participation. Therefore, this study explores whether there are gender differences in the intention to use online learning systems (Ferreira et al., 2018; Mouakket, 2018; Albelali and Alaulamie, 2019). Thirdly, this research selects vocational college students as samples. The majority of past research has concentrated on the fields of higher education (Cranfield et al., 2021; Leo et al., 2021), elementary education, and secondary education (Christakis et al., 2020). In spite of the importance of online learning for students at vocational colleges, most studies conducted during the pandemic ignored this sector.

The purpose of this study is to develop a “continued usage intention model for online learning” that would account for the factors that influence students’ continued usage intentions of online learning systems. These are the sorts of issues that this research begs to address:

1. What factors affect vocational college students’ intention to continue using online learning system?
2. How well do the influencing factors account for the intention to continue using online learning?
3. Are there gender differences in the influencing factors of students’ intention to continue using online learning?

The remaining five portions of the research are as follows: The second part is a literature review of the relevant theory and puts forward the theoretical model of this study as well as hypotheses about the relationships between various variables. The third part describes in detail the methods used in this study and the analysis of the results, followed by a discussion. Finally, the limitations of the study are discussed, along with some recommendations for additional research that could be done in the future.

## 2. Literature review and hypothesis development

### 2.1. Computer anxiety and expectation confirmation model

When faced with a stressful situation, it is normal for people to feel anxious (Fenton et al., 2020).

Anxiety related to using computers is known as “computer anxiety” (CA), which is a form of concept-specific anxiety and a special psychological phenomenon (Saade and Kira, 2006). In this study, “computer anxiety” is defined as a situational fear or anxiety that can be changed when an individual anticipates or actually uses a computer (Sun et al., 2008). The adoption of online learning systems is significantly influenced by anxiety. Individuals who are anxious or unsettled about the prospect of adopting online learning are less likely to use it.

Some researchers have recognized the significance of CA in the intention to use technology continuously (Chou et al., 2012; Purnomo and Lee, 2013; Zarafshani et al., 2020; Seolah et al., 2021). For example, Purnomo and Lee (2013) consider that the fun of playing games on the computer has disappeared when faced with the learning of professional knowledge, which can lead to anxiety and fear in the long run and dampen students’ perceived usefulness of the online learning systems. Hai et al. (2020) pointed out that using new technology tends to evoke more apprehension for users with CA because new technology requires users to learn new terminology, which is more difficult and time-consuming than technology with less complexity. Lu et al. (2019) found that in the online learning environment, students frequently suffer from anxiety when using computers since they are not proficient enough in information technology, fear of poor learning performance, and other reasons. Since perceived usefulness is the most powerful predictor of behavioral intention to use, students with high computer anxiety will indirectly affect their continued use of online learning systems (Niu and Wu, 2022). According to a comprehensive review of the relevant literature (Tri and Hafiz, 2018; Samydevan et al., 2020; Lee and Xiong, 2022), it can be concluded that online learning under the influence of anxiety and fear cannot make them experience the usefulness of online learning (Persada et al., 2021) as well as the unpleasant emotions caused by users’ use of new technologies are unlikely to be alleviated, so it is impossible to form a positive confirmation (Feng et al., 2019). As a result, this research hypothesizes:

*H1:* CA has a negative effect on PU of an online learning system.

*H2:* CA has a negative effect on CON of an online learning system.

### 2.2. Expectation confirmation model

ECM was first proposed by Bhattacherjee (2001), and it is grounded in Oliver’s (1980) expectation confirmation theory (ECT). Perceived usefulness (PU; sometimes called post-adoption expectation), the extent of their confirmation, and satisfaction are the three factors that the ECM posits as determining whether or not users will continue using the technology (Bhattacherjee, 2001; Lee, 2010). Confirmation, when applied to the realm of online learning, refers to the degree to which a learner’s expectations about their online learning experience match their actual online learning experience. According to ECM, after using any technology, users evaluate their performance perception in comparison to their prior expectations and then decide the level of confirmation (Wang and Wang, 2019). Users construct a post-acceptance, also known as a usefulness perception, depending on their usage experience and the level of confirmation. These perceptions of usefulness may differ from or coincide with the users’ initial anticipations. The subsequent formation of satisfaction is caused by the confirmation of expectations and the perception of usefulness (Rahi et al., 2022). Finally, a high degree of technical satisfaction will form a continuous intention (Rabaa'i et al., 2021; Anjum et al., 2022; Huang et al., 2022).

Previous research has demonstrated the relationship between various variables related to ECM (Daneji et al., 2019; Cheng, 2020a,b; Persada et al., 2021; Zuniga-Jara, 2021). Persada et al. (2021) demonstrated that the powerful indicator of users’ continuous intentions is satisfaction. Zuniga-Jara (2021) indicated that perceived usefulness is found to be the most powerful predictor of continuous intention. Bhattacherjee (2001) found that confirmation is the key factor influencing a user’s satisfaction. If online learning systems help users improve their academic performance, they are usually perceived as useful (Baber, 2021; Persada et al., 2021). Confirmation will lead to user satisfaction when users believe it is beneficial and their real use experience matches or surpasses their original expectations (Tan and Kim, 2015; Xu et al., 2017). (Akter et al., 2020) considered that because users’ perceptions of the usefulness of online learning systems can commonly serve as a baseline against confirmation judgments, more useful online learning systems will be more likely to be perceived as satisfactory. Previous empirical studies have also shown that this satisfaction is an important indicator for predicting the intention to continue using online learning systems (Yang, 2018; Chen, 2021; Si et al., 2022). In addition, students who have a positive attitude toward online learning are more likely to use it all the time if they see an improvement in their academic achievement as a result of utilizing the systems (Fang et al., 2017; Wang et al., 2017; Widjaja et al., 2021). Based on a large number of literature analyses, the hypotheses are derived as follows:

*H3:* CON will positively affect SAT with online learning systems.

*H4:* PU will positively affect SAT with online learning systems.

*H5:* PU will positively affect CI of online learning systems.

*H6:* SAT will positively affect CI of online learning systems.

### 2.3. Social interaction and expectation confirmation model

Interaction has been recognized for a very long time as one of the important factors. Although there is no clear definition of interaction at present, its basic feature is behavior involving two or more subjects sharing information and opinions with each other. The definition of interaction in online learning is proposed by various researchers, and it claims that learner–learner, instructor–learner, and content–learner are all possible types of interaction that might take place during online learning (Abrami et al., 2011; Alqurashi, 2018; Baber, 2021). The first two interactions are what we refer to as social interaction. The key to a successful learning experience for students is interactions, which is at the core of the learning process (Keskin et al., 2020).

Numerous studies have examined the link between students’ SI and CI (Lugonzo, 2020; Baber, 2021; Byun et al., 2021; Yoon et al., 2021). For example, using mediator factors like imagery and flow, Rodriguez-Ardura and Meseguer-Artola (2016) discover that the continual learning behavior of students can be encouraged through frequent interaction. Research by Molinillo et al. (2018) demonstrates the positive effects of learner-to-learner and learner-to-instructor interactions on students’ emotional involvement, which in turn enhances students’ active learning and their intention to continue using online learning. Holland (2019) found that when students communicate with each other in online learning, social interaction is fostered by the communication process. Higher levels of satisfaction with online learning are experienced by students who interact more with their teachers and peers. Consequently, social interaction in online learning will enhance the intention to continue using the systems (Panigrahi et al., 2018; Chung et al., 2020; Li and Zhao, 2021). The following is how the hypothesis is formed from an analysis of the available literature:

*H7:* SI will positively affect CI with online learning systems.

### 2.4. Computer self-efficacy and expectation confirmation model

Self-efficacy refers to “the individuals having the ability and belief to complete a specific task, as well as the confidence to cope with future challenges” (Sánchez and Hueros, 2010). In this study, computer self-efficacy is a specific definition of self-efficacy, which refers to the individual’s judgment of their ability to use the online learning system (Venkatesh and Morris, 2000). Individuals who have a higher computer self-efficacy (CSE) magnitude are more likely to believe that they are able to successfully complete challenging computing activities and to believe that they are able to function with a smaller amount of support and assistance than people who have a lower CSE magnitude. In other words, a person’s level of self-efficacy will affect whether or not they participate in activities, how much effort they put into finishing those activities, and how persistent they are in doing so (Bandura and Watts, 1999).

Previous research has suggested that relevant on CSE is linked to CI (Hermawan et al., 2021; Nurhikmah et al., 2021; Putra, 2021). For example, Mushtaque et al. (2022) took 369 newly enrolled medical students as samples to investigate whether or not medical students would like to use the online learning systems during the COVID-19 pandemic. The findings showed that CSE significantly reduced the negative impacts of technological stress and increased the intention to use online learning systems among medical students. Putra (2021) also discovered that CSE had a direct positive influence on students’ continued intention to use online learning. In conclusion, CSE is a critical predictor of the intention to continuously use online learning.

CSE is directly linked to individual confidence in one’s own competence and knowledge in a given area (Hsu et al., 2018; Ren et al., 2018). Relevant research results show that students can feel confident when using online learning systems if they possess the requisite level of competence or knowledge, which helps reinforce the perception of its usefulness (Fathema et al., 2015; Kanwal and Rehman, 2017; Salloum et al., 2019). Thus, students with high CSE may have a higher PU of online learning systems. However, low computer self-efficacy students will experience tension and anxiety when using computers, which will lower their judgment of their usefulness (Rahi et al., 2022). Analysis based on the above literature review, the hypotheses are derived as follows:

*H8:* CSE will positively affect PU with online learning systems.

*H9:* CSE will positively affect CI with online learning systems.

### 2.5. The moderating effect of gender

The literature on social psychology suggests that there are substantial gender-based disparities in how people behave in a variety of decision-making contexts (Bandura, 1986). Meanwhile, it has long been widely accepted that there are significant gender differences in information technology use (Grint et al., 1995; Sandelowski, 2000; Brussevich et al., 2018). A lot of research shows that the online learning motivation of females shows a strong sense of self-guidance and responsibility and that they are more satisfied with the online learning experience (Albelali and Alaulamie, 2019; Malik et al., 2020; Wongwatkit et al., 2020). In addition, some studies have indicated that gender has no influence on online learning satisfaction and learning achievements (Martin and Bolliger, 2018; Alghamdi et al., 2020; Dubois et al., 2020). However, whether gender affects the intentions to use online learning continuously needs to be further clarified.

It has been reported that male and female students may view their own computer self-efficacy differently (Assaker, 2020; Yorulmaz, 2021; Bailey, 2022). Among a group of university freshmen, Yorulmaz (2021) finds that the males are more confident in their abilities to use computers than the females. Recent research into gender disparities in CSE suggests that the difference may be due to the perceived masculinity of the activity in question (Alghamdi et al., 2020; Yorulmaz, 2021). It appears that the gender difference in CSE is caused by the complexity of the task. The perceived masculinity element increases as the activity becomes more difficult, and men demonstrate more self-efficacy for such activities, which in turn affects their intentions to use online learning in a sustained manner. This study thus hypothesizes:

*H10a: There is difference between male and female in CSE and CI.*

According to the available literature, males are more susceptible to utility and expected performance because they are more task-oriented (Assaker, 2020; Pal and Patra, 2021; Kaur and Kaur, 2022). In particular, Kaur and Kaur (2022) found that the correlation between PU and CI is stronger for males than for females, which suggests that gender moderates the relationship. We anticipate that the relationship between PU and CI will be stronger for males in the context of online learning. Males are more task and result-oriented, and they care more concerned with the advantages or utilities they get from using online learning systems. Following this reasoning, the following hypothesis is established:

*H10b:* There is difference between male and female in PU and CI.

The technical aspects of online learning are helpful in accomplishing their learning objectives, thus affecting their SAT with online learning (Brussevich et al., 2018; Alghamdi et al., 2020; Dubois et al., 2020). Therefore, SAT has a greater impact on men’s CI because it depends more on the technical characteristics of online learning than on social characteristics. As males are task-oriented, their SAT with online learning will be more significant. We predict that the difference between men’s and women’s SAT with online learning will affect their CI. The hypothesis is proposed as follows:

*H10c:* There is difference between male and female in SAT and CI.

Males and females are products of two evidently distinct cultures, and as a result, they develop very distinctive approaches to the social interactions appropriate to their respective genders (Wood-Downie et al., 2021). Sian et al. (2020) point out that, in terms of the number of interactions, male students interact more with teachers and peers, and teachers tend to pay more attention to male students, whether it is criticism or encouragement. Males show a more positive attitude in SI, and they are more willing to continue to use online learning. The hypothesis is proposed as follows:

*H10d:* There is difference between male and female in SI and CI.

**Figure 1:**
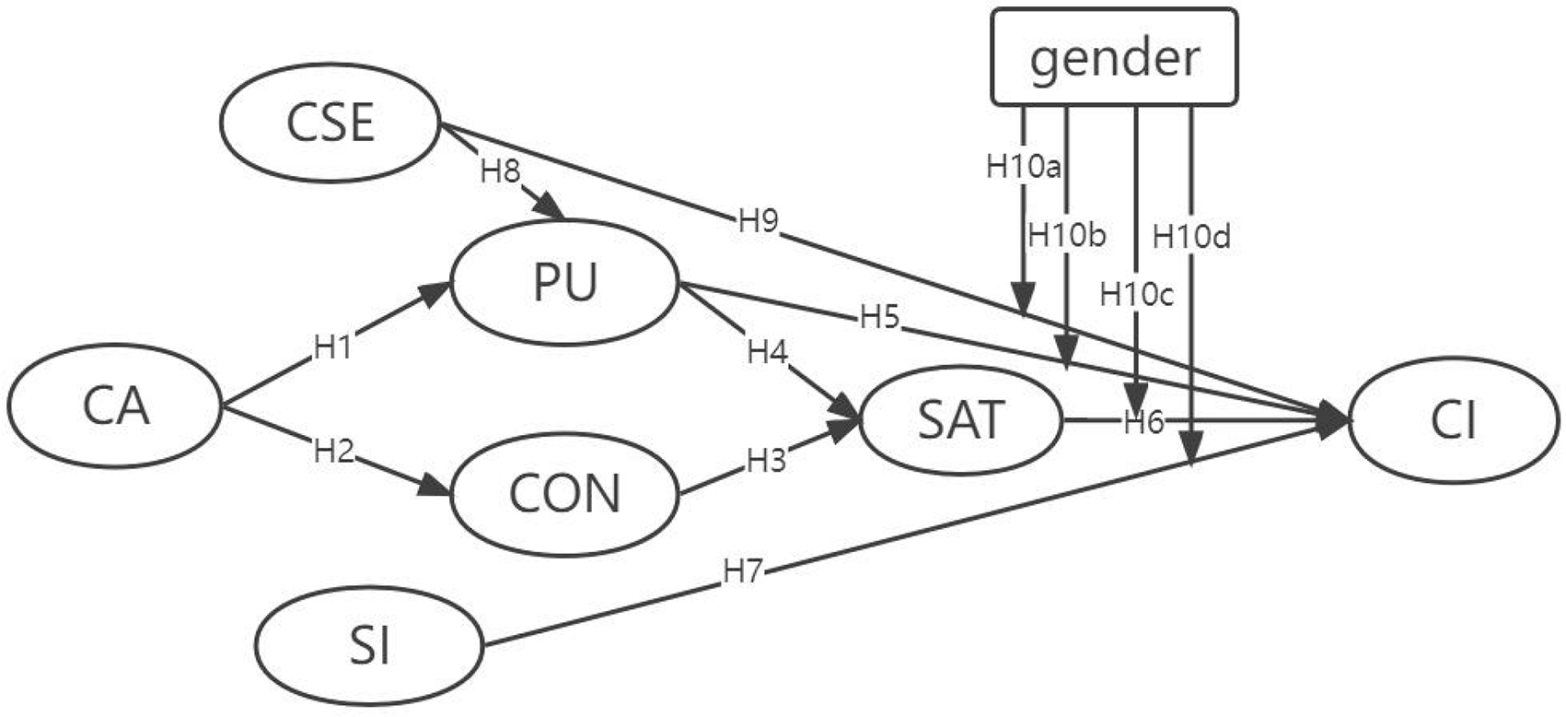
The proposed model of study.

Figure 1 displays all of the hypotheses that are included in the model. Gender is used as a grouping variable to conduct a multi-group analysis of the differences between males and females in vocational college students’ intentions to continue using online learning systems.

## 3. Methodology and materials

### 3.1. Participants

The questionnaire is designed as an online survey with links distributed to the target population through WeChat or email. In the current study, 524 questionnaires were distributed among the students of the eight vocational colleges by using a random sampling method. Therefore, the acquired samples are extremely representative and fit the requirements for this study. A total of 482 valid questionnaires were collected. The descriptive statistics in this study were calculated using SPSS 26.0. There are approximately 243 female respondents, making up 50.4% of the total, as opposed to 239 male respondents, who make up 49.6% of the total (Table 1). The population in the sample ranges in age from 22 to 24 years old the most (36.1%), followed by those aged 18 to 20 years old (34.4%). The students are predominantly humanities students (40.7%). The mean education level is junior in college (27.8% freshmen, 31.5% sophomores, 36.7% juniors).

**Table 1:** Demographic information of the sample.

Characteristics	Item	Frequency	Percentage
Gender	Male	239	49.6%
	Female	243	50.4%
Academic year	Freshman	134	27.8%
	Sophomore	152	31.5%
	Junior	196	40.7%
Major	Humanities major	177	36.7%
	Social science major	138	28.6%
Age	Natural science major	167	34.6%
	18–20	166	34.4%
	20–22	142	29.4%
	22–24	174	36.1%

### 3.2. Instruments

Questionnaires were used to collect information for this study, with the majority of measurement items derived from previously developed scales and modified to fit the current research content. The content design of the questionnaire consists mainly of demographic variables and specific measurement scales. The demographic variables of age, academic year, major, and gender are used to conduct an investigation into the characteristics of the people who took part in this study.

In addition to the demographic information of the participants, the questionnaire consists of seven different constructs, which are presented in Table 2. The questions on the questionnaire used in this study were modified from an earlier study (see Table 2). To measure continued intention, the Chiu and Wang (2008) and Liao et al. (2009) questionnaires are used with three items. Confirmation is assessed with three questions, which are taken from the questionnaire provided by Kang et al. (2009) and Liao et al. (2009). The three-item satisfaction questionnaire developed by Liao et al. (2009) and Chiu and Wang (2008) is used for this research. The questionnaire items on perceived usefulness are adapted from Lee (2006); The items on computer anxiety are from Sun et al. (2008). The items for measuring computer self-efficacy are borrowed from Compeau and Higgins (1995). The social interaction is measured with a four-item scale developed by Molinillo et al. (2018) and Robinson and Hullinger (2008). Every item is rated on a Likert scale of 1–5, with 1 denoting “strongly disagree” and 5 denoting “strongly agree.”

**Table 2:** Questionnaire items.

Construct	Items	Source
Confirmation (CON)	CON1: My experience with using the online learning environment is better than I expected	Kang et al. (2009) and Liao et al. (2009)
	CON2:The service level provided by the online learning environment exceeds my expectations	
	CON3: Overall, the majority of my expectations for the online learning environment have been confirmed	
Continue intention (CINT)	CINT1: I intend to continue using the online learning in the foreseeable future	
	CINT2: I will continue using the online learning in the future	
		Chiu and Wang (2008) and Liao et al. (2009)
	CINT3: I will regularly use the online learning in the future	
Satisfaction (SAT)	SAT1: I am satisfied with the performance of the online learning	Chiu and Wang (2008) and Liao et al. (2009)
	SAT2: I am delighted with the experience of using the online learning	
	SAT3: My decision to use the online learning is a rational one	
Perceived usefulness (PU)	PU1: Using an online learning system improves my learning effectiveness	Lee (2006)
	PU2: Using the online learning system will enhance my learning performance	
	PU3: I believe online learning contents are informative	
Computer anxiety (CA)	CA1: Working with a computer is something that would give me a lot of anxiety	Sun et al. (2008)
	CA2: I get depressed when I think of trying to use a computer	
	CA3: Computers make me feel uneasy and confused	
	CA4: I feel apprehensive about using the computer system	
Social interaction (SI)	SI1: The teacher or instructor of an online learning system fosters a positive environment and makes opportunities for students to interact with one another	Molinillo et al. (2018) and Robinson and Hullinger (2008)
	SI2: The online learning system teacher/instructor encourages communications between learners and teachers	
	SI3:I communicate with other students while studying online	
	SI4: I converse and share opinions with other students during online learning	
Computer Self-efficacy (CSE)	CSE1: Despite the fact that nobody was around to direct me, I feel confident working on the computer	Compeau and Higgins (1995)
	CSE2: I’m competent at resolving computer issues	
	CSE3:I’m not nervous about using software I’ve never used before	

### 3.3. Procedure

To begin, eight vocational colleges were chosen at random in the province of Jiangxi, and the informed consent form was distributed to the teachers in charge of each major with the approval of the principals of each school. Secondly, after obtaining the teacher’s permission, students from vocational colleges of all majors will take the main test. At the same time, under the premise of firmly ensuring that responses are kept anonymous, the electronic questionnaire is made available to students by means of the platform provided by www.wjx.com. Finally, when the questionnaire is distributed, the requirements for filling in the questionnaire and precautions are explained. In addition, guide the filling of the questionnaire in a timely manner, and make it clear that the data will never be used for anything other than study in the academic sphere. The data was gathered between August 10 and October 5, 2022.

## 4. Results

The data is analyzed with the assistance of the Smart-PLS 3.0 software (Ringle et al., 2015), which is a multivariate method that belongs to the second generation. This requires conducting an analysis in two stages, which are as follows: (1) the measurement models (2) the structural model or inner model.

### 4.1. Measurement model assessment

Construct reliability, convergent validity, and discriminant validity are estimated to determine the measurement model’s overall quality in this research.

Cronbach’s alpha and composite reliability are used to assess the internal consistency of multi-item scales, guaranteeing that all items on the scale are measuring the same construct. As can be seen from Table 3, all of the CA and CR values for the constructs are greater than the minimum threshold of 0.7, indicating a high degree of reliability (Holmes-Smith, 2001).

**Table 3:** Measurement model assessment.

Dimension	Items	CR	AVE	CA
CI	CI1	0.886	0.598	0.943
	CI2			
	CI3			
CON	CON1	0.852	0.603	0.937
	CON2			
	CON3			
SAT	SAT 1	0.850	0.645	0.940
	SAT 2			
	SAT 3			
PU	PU1	0.838	0.589	0.934
	PU2			
	PU3			
CA	CA1	0.879	0.645	0.929
	CA2			
	CA3			
	CA4			
CSE	CSE1	0.875	0.579	0.885
	CSE2			
	CSE3			
SI	SI1	0.898	0.622	0.879
	SI2			
	SI3			

CA, Cronbach’s alpha; CR, composite reliability; AVE, average variance extracted; CI, continuance intention; CON, confirmation; SAT, satisfaction; PU, perceived usefulness; CA, computer anxiety; CSE, computer self-efficacy; SI, social interaction.

Convergent validity denotes the degree of correlation between the factor loadings of a variable. According to (Hair et al., 2016), Since AVE is more than 0.5, it may be deduced that the convergent validity of the variables meets the requirements. The findings presented in Table 3 provide evidence that the constructs have satisfactory levels of convergent validity.

The discriminant validity should be examined to estimate the degree to which one concept and its indicators are distinct from those of another concept and their indicators (Bagozzi et al., 1991). It could.

be evaluated by the Fornell-Larcker scale (i.e., the square root of AVE) and the Heterotrait-Monotrait ratio of correlations (HTMT). According to Fornell and Larcker (1981), the first criterion for discriminating validity is that the correlation between items in any two structures should be less than the square root of the AVE shared by items within a construct. The results in Table 4 demonstrate sufficient discriminant validity.

**Table 4:** Fornell-Larcker.

	CA	PU	CON	SAT	SE	SI	CI
CA	**0.796**						
PU	0.523	**0.875**					
CON	0.488	0.433	**0.854**				
SAT	0.566	0.322	0.336	**0.788**			
CSE	0.476	0.565	0.422	0.354	**0.841**		
SI	0.514	0.478	0.534	0.409	0.563	**0.886**	
CI	0.204	0.534	0.421	0.331	0.452	0.426	**0.798**

The diagonal values in bold are the square roots of AVE.

The HTMT serves as the second criterion for evaluating discriminant validity. It is the ratio of the mean value of the index correlation between different constructs to the mean value of the index correlation between the same constructs (Henseler et al., 2016). Markus (2012) propose that the HTMT should be <0.85, indicating good discrimination validity. The findings are presented in Table 5, which demonstrates that every value meets the requirement.

**Table 5:** HTMT.

	CA	PU	CON	SAT	SE	SI	CI
CA	0.456						
PU	0.223	0.305					
CON	0.188	0.287	0.424				
SAT	0.266	0.322	0.336	0.388			
CSE	0.176	0.265	0.222	0.354	0.291		
SI	0.014	0.278	0.334	0.209	0.163	0.286	
CI	0.204	0.134	0.121	0.331	0.252	0.226	0.198

In conclusion, the measurement models have high reliability, discriminant validity, and convergent validity, which can be used to evaluate the structural model.

### 4.2. Structural model assessment

For structural models, the important evaluation indices are the coefficient of determination (*R*^2^), predictive accuracy (*Q*^2^), collinearity, model fit, and the size of the structural path coefficient and its statistical significance.

#### 4.2.1. Collinearity test

Concerning the structural model, it is essential to perform an analysis to determine whether or not there is collinearity between the independent variables. The variance inflation factor (VIF) is used to investigate whether there is collinearity among the predictor constructs, which must be <5 (Sarstedt et al., 2011). The findings shown in Table 6 demonstrate that the VIF values are appropriate.

**Table 6:** Collinearity test.

	CA	PU	CON	SAT	SE	SI	CI
CA		2.091	3.788				
PU				3.599			4.056
CON				2.786			4.623
SAT							3.886
CSE		3.865					3.666
SI							2.674
CI							

#### 4.2.2. Coefficient of determination (*R*^2^)

The method to evaluate the explanatory power of structural models uses the coefficient of determination (*R*^2^). A latent variable’s *R*^2^ value is a measurement of the relationship between its explained variance and its total variance. Values around 0.670 are regarded as substantial, 0.333 as moderate, and 0.190 as weak (Chin, 1998). In the research, the *R*^2^ value of the intentions to continue using online learning systems reached 0.766 (substantive). In addition, the *R*^2^ values of satisfaction, perceived usefulness, and confirmation also reached above the medium level (Table 7).

**Table 7:** Coefficient of determination.

Construct	*R* ^2^	Results
PU	0.552	Moderate
CON	0.501	Moderate
SAT	0.522	Moderate
CI	0.766	Substantive

#### 4.2.3. Predictive relevance

This study uses Stone-Geisser’s cross-validation method to calculate the *Q*^2^ value in order to analyze the predictive relevance of the model (Vinzi et al., 2010). Table 8 shows that *Q*^2^ values are >0, so the constructs should be acknowledged as having sufficient predictive relevance (Hair et al., 2019a).

**Table 8:** Predictive accuracy.

Construct	*Q* ^2^
PU	0.595
CON	0.345
SAT	0.486
CI	0.620

#### 4.2.4. Absolute model fit indices

Model fit can be evaluated with the use of a statistic known as the Standardized Root Mean Square Residual (SRMR; Nayernia, 2020). According to Table 9, the SRMR value of 0.058 that was calculated using Smart-PLS 3 is lower than the required value of 0.08, which further demonstrates that the overall model developed for this investigation exhibits a level of fit that is acceptable (Cepeda-Carrion et al., 2018; Hair et al., 2019b).

**Table 9:** Model fit.

Construct	Acceptable value	Actual value
SRMR	<0.08	0.065

#### 4.2.5. Hypothesis tests

In this part of the study, the research hypothesis is tested, which revolves around the relationship between constructs. The relationship will be considered statistically significant if the values of the *t*-statistic are higher than 1.96. Path coefficients are used in the analysis process in order to determine the influence of each independent component on the dependent variable. Therefore, the value of the path coefficient determines the extent to which it has an effect, and the larger its value, the more significant its effect. Table 10 displays a summary of the results of the hypothesis.

**Table 10:** Summary of hypothesis results.

Hypotheses	Path coefficient	Standard error	*T*-Value	Value of *p*	Test result
H1	0.133	0.045	2.956	0.000^***^	Supported
H2	0.711	0.053	13.415	0.000^***^	Supported
H3	−0.454	0.063	7.206	0.000^***^	Not Supported
H4	0.071	0.035	2.029	0.006^**^	Supported
H5	0.664	0.047	14.128	0.000^***^	Supported
H6	0.332	0.046	7.217	0.000^***^	Supported
H7	0.141	0.053	2.660	0.005^**^	Supported
H8	0.372	0.056	6.642	0.000^***^	Supported
H9	0.432	0.051	8.470	0.000^***^	Supported

In terms of the first hypothesis, CA and PU have a positive and statistically significant correlation (*β* = 0.133, *t* = 2.956, *p* < 0.001). The second hypothesis is also supported by the data, which show a positive relationship between CA and CON (*β* = 0.711, *t* = 13.415, *p* < 0.001). The third hypothesis is negatively supported, between CON and SAT (*β* = −0.454, *t* = 7.206, *p* < 0.001). The fourth hypothesis assumes a positive and statistically significant relationship between PU and SAT (*β* = 0.071, *t* = 2.029, *p* < 0.05). PU and CI demonstrate a significant association (*β* = 0.664, *p* < 0.001), supporting hypothesis 5. The sixth hypothesis asserts that there is a positive and significant link between SAT and CI (*β* = 0.322, *t* = 7.217, *p* < 0.001). The relationship between SI and CI is significant (*β* = 0.141, *t* = 2.660, *p* < 0.05), which supports the seventh hypothesis. There is a significant relationship between CSE and PU (*β* = 0.372, *t* = 6.642, *p* < 0.001), which supports the eighth hypothesis. The hypothesis states that there is a strong positive correlation between CSE and CI (*β* = 0.432, *p* < 0.001), supporting hypothesis 9.

### 4.3. Multi-group analysis

There are three stages to a multi-group analysis: Data groups are generated in Step 1, and MICOM analysis is performed in Step 2 using the standard three-step process. Step 3 involves evaluating the outcomes of multi-group comparison statistical tests.

The first step is to form a data set. Aiming to evaluate dissimilarities among different groups. Groups are divided into males and females according to the purpose of the study (Lohmöller, 1989). However, results from statistical tests may be skewed, if the disparity in sample sizes between the two groups is >50% (Hair Jr. and Page 2015). There are 239 men and 243 females, respectively, representing a difference of <50%. Consequently, the statistical findings have no bias.

Secondly, Henseler et al. (2016) state that it is essential to carry out the MICOM before performing the multigroup analysis. The purpose of this MICOM is to provide empirical evidence to support that the difference between the two groups is due to the structural model rather than the measurement model (Henseler et al., 2016).

There are three steps involved in MICOM: (1) ensuring configuration invariance; (2) ensuring compositional invariance; and (3) ensuring the composite mean values and variances are equal. Firstly, diverse groups use totally consistent measurement items, methods for processing data, and approaches for analyzing data. In light of this, the data for the male and female groups has been established for configuration invariance. Secondly, a permutation test is used to determine whether or not compositional invariance exists. The compositional invariance test requires that the original correlations should be equal to or greater than the 5.00% quantile correlations. The last step is to determine whether or not the variances and mean values of the groups are equal. According to Table 11, compositional invariance exists.

**Table 11:** Measurement invariance test using MICOM.

	Original correlation	5.00%	Permutation *p*-value	Results
CA	1.000	1.000	0.838	Yes
PU	1.000	1.000	0.833	Yes
CON	1.000	1.000	0.175	Yes
SAT	0.998	0.998	0.359	Yes
CSE	1.000	1.000	0.412	Yes
SI	1.000	1.000	0.263	Yes
CI	1.000	0.999	0.161	Yes

The difference between the composite’s mean and variance ratio results must fall within the 95% confidence interval. Tables 12, 13 both show evidence of partial invariance. Actually, PLS-MGA can be used for multi-group analysis to compare the structural paths between groups even when only partial measurement invariance is present (Henseler et al., 2016).

**Table 12:** MICOM Step 3 results report—part 1.

	Mean-original difference (male–female)	Mean-permutation mean difference (male–female)	0.025	0.975	Permutation *p*-values	Equal mean values
CA	0.468	0.002	−0.253	0.262	0.001	NO
PU	0.385	0.002	−0.245	0.271	0.005	NO
CON	0.332	0.003	−0.260	0.263	0.014	NO
SAT	0.448	0.002	−0.255	0.245	0.000	NO
CSE	0.413	0.003	−0.256	0.272	0.006	NO
SI	0.369	0.002	−0.252	0.265	0.002	NO
CI	0.455	0.002	−0.246	0.267	0.000	NO

**Table 13:** MICOM Step 3 results report—part 2.

	Variance-original difference (male–female)	Variance-permutation mean difference (male–female)	0.025	0.975	Permutation *p*-values	Equal mean values
CA	0.468	0.002	−0.252	0.232	0.001	NO
PU	0.225	0.002	−0.225	0.261	0.227	Yes
CON	0.332	0.003	−0.260	0.251	0.014	NO
SAT	0.233	0.002	−0.254	0.245	0.322	Yes
CSE	0.413	0.003	−0.226	0.242	0.003	NO
SI	0.369	0.002	−0.255	0.275	0.001	NO
CI	0.455	0.002	−0.241	0.237	0.000	NO

Finally, to test if significant statistical differences exist between males and females, we employ the PLS-MGA. The path coefficient and mean difference of the composite are shown in Table 14. At the same time, the study reveals that male and female data sets have distinct differences in the impact of perceived usefulness on the intention to continue using online learning systems. Other path hypotheses show no significant difference (Table 15).

**Table 14:** Assessment of group differences.

	Path coefficients original (males)	Path coefficients original (females)	Path coefficients original difference (males–females)	2.5%	97.5%	Permutation *p*-value	Supported
CSE → CI	0.765	0.626	0.139	−0.211	0.202	0.415	NO
SI → CI	0.063	0.186	−0.123	−0.299	0.284	0.241	NO
PU → CI	0.012	0.023	−0.116	−0.113	0.115	0.000	Yes
SA → CI	0.370	0.567	−0.206	−0.377	0.201	0.261	NO

**Table 15:** Comparison analysis.

	Path coefficient	Value of *p* (female)	Results	Path coefficient	Value of *p* (male)	Results
CSE → CI	0.358	0.000	Accepted	0.288	0.000	Accepted
SI → CI	0.552	0.007	Accepted	0.554	0.005	Accepted
PU → CI	0.466	0.001	Accepted	0.443	0.000	Accepted
SAT → CI	0.485	0.000	Accepted	0.376	0.000	Accepted

## 5. Discussion

Due to the rapid spread of the pandemic, there was a drastic transition in teaching methods, with traditional classroom education was supplanted by education delivered *via* the internet in a relatively short amount of time (Mushtaque et al., 2021).

The goal of this study is to determine what elements influence vocational college students’ intention to continue using online learning systems. Furthermore, this study also explores whether there is a gender difference among these factors in light of the growing interest in online learning. In order to better understand the causal mechanism of students’ intention to continue using online learning, this study constructs a model to explore the causal relationship between variables.

The research results could fall into two distinct parts. First, this study sheds light on the relationships between the ECM variables. Results from our analysis show that in addition to the significant negative correlation between CON and SAT, other hypotheses about the relationship between ECM variables PU, SAT, CON and CI are supported. It’s clear that our findings align with those of Mushtaque et al. (2022), which conclude that SAT, CON, and PU all have a role in shaping vocational students’ continuation intentions toward online learning systems. The following is a detailed description:

There is a negative relationship between CON and SAT. These findings are inconsistent with preceding ECM-based studies, as they reported that learners’ initial expectations of the online learning system are positive predictors of satisfaction (Chong, 2021; Barroso et al., 2022; Si et al., 2022). It’s likely that people’s expectations for the system are based on their previous experiences with similar systems, and this could be one reason. Due to the impact of the epidemic, the traditional face-to-face learning mode has changed to online learning, while vocational college students lack online learning experience.

It is possible that the correlation between PU and SAT can be explained by the fact that when students’ performance is improved through the use of an online learning system, they typically demonstrate high levels of satisfaction. This finding is unanimous with the outcomes of prior research (Chen and Keng, 2019; Ashraf et al., 2020; Soria-Barreto et al., 2021). As reported by Olasina (2018), students pay greater attention to their individual needs while using online learning systems. Students may be satisfied with online learning systems if they have the perception that those systems can enhance their capabilities, work performance, or the effectiveness of their learning.

The empirical findings confirm the considerable impact of SAT on CI in online learning settings.

Previous research has found the same results (Panigrahi et al., 2018; Chung et al., 2020; Yoon et al., 2021). Satisfaction is related to learners’ learning experiences. According to the research conclusion, learners’ learning experiences are highly correlated with their computer anxiety during the learning process. Therefore, reducing the difficulty of online learning systems and improving their self-confidence are particularly important for promoting learners’ intentions to continue to participate in online learning.

The findings show that students’ intentions to continue using online learning systems are significantly impacted by PU. The results corroborate those of earlier research (Bawack and Ahmad, 2021; Han and Du, 2021; Barroso et al., 2022). The perception of usefulness is mainly reflected in the fact that using online learning systems to learn can improve learning effects and efficiency, and the improvement of usefulness will directly affect their intentions to continue using online learning systems to learn in the future.

Second, the findings have demonstrated that all of the hypotheses pertaining to the relationships between the various external variables and PU, SAT, CON, and CI are supported.

This study found that CA has a negative impact on PU. This suggests that perceived usefulness decreases as anxiety levels increase. These results are consistent with what was found by Chang et al. (2017) and Wu and Wang (2020). With the continuous accumulation of experience in using online learning systems, users’ perception of anxiety will also be reduced. Teachers should therefore focus more on and mentor students with limited computer abilities in online learning to help them achieve better, so as to improve their perception of the usefulness of online learning.

The results of this study show that anxiety has a prominent influence on vocational college students’ confirmation. Having anxiety makes it more difficult to confirm, as the relationship is negative. Previous research has supported this relationship (Lee et al., 2009; Rabaa'i et al., 2021; Barroso et al., 2022). The anxiety generated by students in the online learning process will inhibit the positive expectations of the system.

Prior researches points out to that CSE is a critical factor in determining whether someone chooses to use computers (online learning systems) through PU (Mouakket and Bettayeb, 2015; Eraslan Yalcin and Kutlu, 2019; Lew et al., 2019). It can provide students with additional computer use opportunities to help them increase their future use and improve their self-efficacy.

This research demonstrates that SI has a significant role in determining CI. In light of this, it can be deduced that SI is still an important component of effective online learning, which is consistent with the findings of Eom and Ashill (2016); (Eom et al., 2010; Lasfeto, 2020; Mehall, 2020). People are unable to leave their homes or participate in any outside social activities while the blockade is in place. One of the benefits of online learning is the increased potential for students to interact with teachers, classmates, and people from other parts of the world. If potential users of online learning systems hear from others in their social environment that the online learning system is simple to operate, they may believe that switching to the online learning system will require little effort.

The findings also indicate that a student’s CSE has a positive correlation with their CI to use online learning systems. As a consequence of this, the higher the CSE of the students, the higher their intention to continue using online learning systems. The finding is identical with the results of Nurhikmah et al. (2021), who discovered that higher CSE contributes to higher computer usage. The findings, however, show that CSE is the weakest predictor. When students grow more and more familiar with online learning systems, the significance of CSE may diminish.

Remarkably, MGA results indicate that there is a remarkable difference between males and females in the relationship of PU to CI. There is no significant difference in other path assumptions. However, this difference does not exist naturally. It overturns the conventional understanding that males have natural advantages in the use of technology (Fu et al., 2022). From the perspective of the path coefficient (PU → CI), females are higher than males. This shows that females pay more attention to the classroom teaching and teaching evaluation of online learning than boys and have a more significant impact on their intentions to continue using online learning. In addition, females pay more attention to the influence of PU than males, which also shows that females always maintain the gender impression of “hard work” in learning and that online learning has stronger endogenous motivation.

In conclusion, the findings of this study suggest that ECM can be applied to assess the students’ intention to continue utilizing an online learning system. Nevertheless, it is essential to note that students place varying amounts of importance on the factors that determine CI but place less emphasis on CSE. In addition, we ought to pay attention to the moderating effect of gender on students’ intentions to use online learning continuously.

## 6. Implications

### 6.1. Theoretical implications

This research takes students from eight vocational colleges in Jiangxi Province as the research object to explore the influencing factors of online learning’s intention to use continuously. The research conclusion has certain theoretical contributions.

Firstly, innovation in research perspectives. This paper discusses the influencing factors and the interaction mechanism between the vocational college students’ intention to use online learning continuously, filling the gap that most current relevant researches focus on general college education and ignore vocational college students (Niu et al., 2022).

The second is the innovation of research variables. In previous studies based on an expectation confirmation model to reveal learners’ intention to use continuously, the independent variables were mostly external factors (Wang T. et al., 2021). This research creatively introduces emotional factors such as computer anxiety and self-efficacy as independent variables in the process of online learning expectation confirmation. It is helpful to promote the innovation of research conclusions by mining variables that promote students’ continuous use of online learning from their own factors.

Third, innovation of research focus. In the past, most studies on the intention to continue using online learning treated samples as homogeneous groups (Wu and Wang, 2018; Wu and Tian, 2021), ignoring differences among users. This study is conducted from the perspective of gender, and the results show that gender moderates the relationship between perceived usefulness and intention to continually use, while having no significant impact on other paths, which is conducive to the promotion of research conclusions.

### 6.2. Practical implications

In the post-epidemic era, online learning will become a prevalent form of learning, and one important research direction is to clarify how to enhance students’ continuous use intentions. Accordingly, this study proposes the following practical implications based on the results:

From the perspective of system developers, there are numerous approaches to enhance the design of online learning systems, which include enriching the systems’ course content, optimizing the functional layout, making the systems’ navigation function clearer, increasing students’ search efficiency, and streamlining the use of the platform’s interactive functions and tools. In order to further enhance the quality of the material presented in the online course, the system developers need to change the course design based on the learning behavior data of the students and provide meaningful course material to boost students’ perception of the usefulness of the system.

From the perspective of educators, teachers should cultivate students’ online learning abilities, such as the capacity to conduct an efficient search for useful educational resources and to make appropriate use of a wide range of educational tools. Only when students become proficient in a variety of learning strategies who can their sense of self-efficacy be further improved. Additionally, effective online learning relies heavily on social interaction. Therefore, it is necessary to design realistic ways to improve student-teacher and student–student interaction. The instructional design of online classrooms may incorporate collaborative course activities such as group work and debate, as well as other forms of communication tools, such as chat rooms and discussion boards, to encourage student engagement and interaction. Meanwhile, teachers should also avoid stereotypes and recognize that, under the same educational background, the difference in the intentions of males and females to continue to use online learning is gradually narrowing.

## 7. Limitations

The findings of this study have a great deal of important repercussions. Nevertheless, there are also certain limits, which will be discussed in more detail below. First of all, only students enrolled in vocational colleges are included in the sample for this research. In the future, researchers can investigate whether or not there are differences in what ways by comparing vocational college students with general higher education students, with the goal of better understanding the vocational college students’ intention to continue using online learning. Secondly, the conclusions are drawn solely from statistical data, which might make it more difficult to have an in-depth discussion about the continued use of online learning in emergency management. Therefore, future research can include qualitative methods, which could help find more key elements of the continued intention, and the results might help to explain deficiencies in quantitative research. Finally, the focus of this study is on the intention to continue using online learning system, the ultimate objective of all study on intention is to forecast and account for behavior in essence. Previous studies have shown that there is a significant correlation between the intention to continue using online learning and behavior. Future studies can further explore whether intention and behavior are directly related, indirectly related, or affected by other factors.

## 8. Conclusion

This study aims to explore the influencing factors of vocational college students’ intentions to continue using online learning systems in the post-epidemic era. This research outcomes reveal that the expansion model based on ECM by social interaction, computer anxiety, and computer self-efficacy can well predict the continuous use intention of students’ online learning. In addition, there are differences between males and females in the influence of perceived usefulness on the intention to continue using the online learning systems, while gender does not have a substantial effect on the moderation of other paths. This research provides new insights for studies based on the intention to use online learning continuously in recent years. At the same time, we also put forward reasonable and feasible suggestions to the system developers of the online learning system and teachers so that they can optimize the platform functions and enhance students’ online experience from the perspective of students in the process of platform development and teaching so as to ultimately promote students’ intentions to continue to use online learning.

## Data availability statement

The raw data supporting the conclusions of this article will be made available by the authors, without undue reservation.

## Author contributions

CW: conceptualization. XL and XW: data curation. XL: writing original draft. XL, XW, and CW: writing–review and editing. All the authors have read and approved to the published version of the manuscript.

## Conflict of interest

The authors declare that the research was conducted in the absence of any commercial or financial relationships that could be construed as a potential conflict of interest.

## Publisher’s note

All claims expressed in this article are solely those of the authors and do not necessarily represent those of their affiliated organizations, or those of the publisher, the editors and the reviewers. Any product that may be evaluated in this article, or claim that may be made by its manufacturer, is not guaranteed or endorsed by the publisher.
